# Conjugation of Penicillin-G with Silver(I) Ions Expands Its Antimicrobial Activity against Gram Negative Bacteria

**DOI:** 10.3390/antibiotics9010025

**Published:** 2020-01-13

**Authors:** Ioannis Ketikidis, Christina N. Banti, Nikolaos Kourkoumelis, Constantinos G. Tsiafoulis, Christina Papachristodoulou, Angelos G. Kalampounias, Sotiris K. Hadjikakou

**Affiliations:** 1 Inorganic Chemistry laboratory, Department of Chemistry, University of Ioannina, 45110 Ioannina, Greece; giannhsketikidis@yahoo.gr; 2 Medical Physics Laboratory, Medical School, University of Ioannina, 45110 Ioannina, Greece; nkourkou@uoi.gr; 3 Laboratory of Analytical Chemistry, Department of Chemistry, University of Ioannina, 45110 Ioannina, Greece; 4 Department of Physics, University of Ioannina, 45110 Ioannina, Greece; xpapaxri@uoi.gr; 5 Physical Chemistry Laboratory, Department of Chemistry, University of Ioannina, 45110 Ioannina, Greece; akalamp@uoi.gr; 6 Institute of Materials Science and Computing, University Research Center of Ioannina (URCI), 45110 Ioannina, Greece

**Keywords:** biological inorganic chemistry, Diffusion-Ordered NMR Spectroscopy (DOSY-NMR), modified antibiotic, penicillin, antimicrobial activity, toxicity

## Abstract

Conjugation of penicillin G (**PenH**) with silver(I) ions forms a new CoMeD (conjugate of metal with a drug) with formula [Ag(pen)(CH_3_OH)]_2_ (**PenAg**). **PenAg** was characterized by a plethora of physical and spectroscopic techniques, which include in the solid state m.p.; elemental analysis; X-ray fluorescence (XRF) spectroscopy; scanning electron microscopy (SEM); energy-dispersive X-ray spectroscopy (EDX); FT-IR; and in solution: attenuated total reflection spectroscopy (FT-IR-ATR), UV–Vis, ^1^H NMR, and atomic absorption (AA). The structure of **PenAg** was determined by NMR spectroscopy. Silver(I) ions coordinate to the carboxylic group of **PenH**, while secondary intra-molecular interactions are developed through (i) the nitrogen atom of the amide group in MeOD-d_4_ or (ii) the sulfur atom in the thietane ring in deuterated dimethyl sulfoxide DMSO-*d_6_*. The antibacterial activities of **PenAg** and the sodium salt of penicillin (**PenNa**) (the formulation which is clinically used) against Gram positive (*Staphylococcus epidermidis* (*S. epidermidis*) and *Staphylococcus aureus* (*S. aureus*)) and Gram negative (*Pseudomonas aeruginosa* (*P. aeuroginosa PAO1*)) bacteria were evaluated by the means of minimum inhibitory concentration (MIC), minimum bactericidal concentration (MBC), and inhibition zone (IZ). **PenAg** inhibits the growth of the Gram negative bacterial strain *P. aeuroginosa* with a MIC value of 23.00 ± 2.29 μM, in contrast to **PenNa**, which shows no such activity (>2 mM). The corresponding antimicrobial activities of **PenAg** against the Gram positive bacteria *S. epidermidis* and *S. aureus* are even better than those of **PenNa**. Moreover, **PenAg** exhibits no in vivo toxicity against *Artemia salina* at concentration up to 300 μΜ. The wide therapeutic window and the low toxicity, make **PenAg** a possible candidate for the development of a new antibiotic.

## 1. Introduction

Penicillins belong to the group of beta-lactam antibiotics, members of which each contain a high-energy, four-membered beta-lactam ring. This ring is required for exhibiting antibacterial activity. Beta-lactam antibiotics inhibit the synthesis of bacterial cell membranes, while they activate the autolytic enzymes which destroy cell membranes, simultaneously [1]. Nowadays, penicillins constitute about 50% of the antimicrobial agents currently in use (WHO 2018a, 2018b) [2] and they are first choice drugs in the treatment of nosocomial infections [2]. Benzyl-penicillin (penicillin G) is the gold standard penicillin, which is obtained biotechnologically using the fungus *P. chrysogenum* [1]. However, benzyl-penicillin has a narrow antimicrobial spectrum, considering that it is active only towards Gram-positive bacteria (e.g., *Staphylococcus*), and not Gram-negative ones [1]. Moreover, due to wide use of penicillins, infections caused by penicillin-resistant bacteria are hardly treated. Therefore, in order to overcome this problem, new β-lactam compounds with wider spectra of activity and less susceptibility to penicillinases should be developed [3].

Silver ions, on the other hand, have been used as antimicrobial agents for centuries for the prevention and treatment of bacterial infections. In 2009, the National Health Service spent around £25 million on silver-containing dressings and it has been estimated that 15 metric tonnes of silver were incorporated into medical products worldwide in 2010 alone [4]. Silver cations permeate the microbial membranes, bind to DNA, proteins, and free thiol groups of cysteine residues. They also interfere with electron transport, and/or the membrane ion-exchange systems. Due to poor bioavailability, on the other hand, silver cations exhibit low toxicity to mammalian cells, increasing their appeal for clinical use as antimicrobial agents (for chronic wounds and burn infections) [5]. Moreover, the bacterial resistance to silver remains low due to the nonspecific nature of silver’s toxicity [5].

The conjugation of metals with drugs (CoMeDs) is a new research area for the discovery of new synergistic therapeutic modalities. CoMeDs combines two different agents, such as silver(I) with penicillin to create new agents with new properties from the superimposition of those initially possessed by the constituents [6,7,8,9,10,11,12]. Although the term “conjugate” is more properly used for polymer-drugs or dendrimer-drug conjugates where nanoparticles are formed, recently, the term CoMeD has also been used to emphasize the synergistic effect of the coordination of a metal with drugs [13,14]. Furthermore, the solvent type affects the structure of the metal-drug complexes, leading to the formation of polymeric structures by the metal–drug–solvent conjugation.

**Scheme 1:**
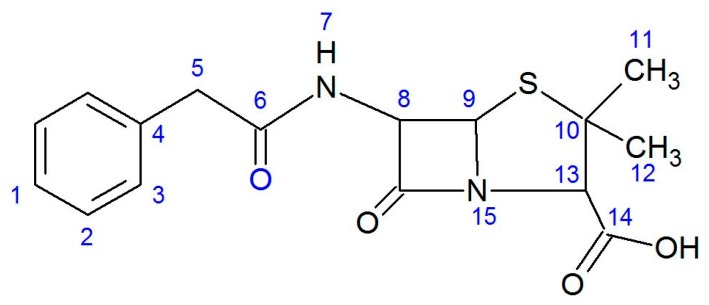
Molecular structure of Penicillin G.

Aiming to develop new antibacterial CoMeDs with little to no bacterial resistance, a new silver(I) conjugate with penicillin G (**PenH** (Scheme 1)) [Ag(pen)(CH_3_OH)]]_2_ (**PenAg**) was synthesized. **PenAg** was characterized by a plethora of physical and spectroscopic techniques; in solid state: m.p., e.a., FT-IR, SEM-EDX, XRF, and XPRD; and in solution: FT-IR, UV–Vis, ^1^H NMR, and HRMS. The antibacterial agent **PenAg** was tested against Gram-positive *Staphylococcus epidermidis* and *S. aureus* and Gram-negative *Pseudomonas aeruginosa* (*PAO1*) bacteria by the means of minimum inhibitory concentration (MIC), minimum bactericidal concentration (MBC), and inhibition zone (IZ). The in vivo toxicity of **PenAg** was tested against *Artemia salina*.

## 2. Results

### 2.1. General Aspects

**Scheme 2:**
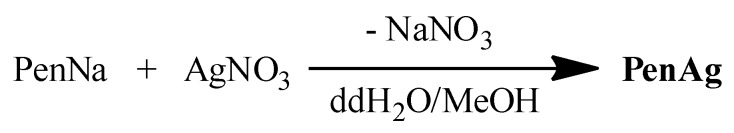
**PenAg** synthetic route.

**PenAg** was obtained by reacting equimolar amounts of silver nitrate with **PenNa** in methanol/water solution (Scheme 2). The elemental analysis reveals that the content of **PenAg** in silver is (21.35 ± 2.33)%, while the calculated one for [Ag(pen)(CH_3_OH)]_2_ is 22.79%. Many attempts to growth crystals of **PenAg** from a variety of solvents and techniques were performed unsuccessfully. The complex is soluble in methanol, acetonitrile, acetone, dichloromethane, dimethylformamide, and dimethylsulfoxide.

### 2.2. Characterization of **PenAg** in Solid State Studies

*X-ray fluorescence spectroscopy:* The XRF spectrum of **PenAg** powder confirms the presence of Ag, while the content of silver found was determined to be 19.0% ± 3.0% *w*/*w*. This is in accordance with the calculated one for the formula [Ag(pen)(CH_3_OH)]_2_ (22.79% *w*/*w*). The seeming discrepancy between the experimentally obtained Ag content and the calculated one is in the frame of the accuracy of the method. Moreover, the standard deviation in the silver analysis with XRF spectroscopy (±3.0% *w*/*w*), is within the calculated content in silver.

**Figure 1:**
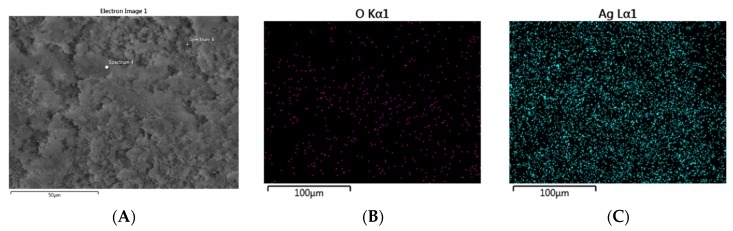
SEM image accompanied by EDX qualitative analysis. The dot marks the location of EDX (**A**). EDX chemical maps (**B**–**C**).

*Scanning electron microscopy (SEM) and Energy-dispersive X-ray spectroscopy (EDX):* The elemental analysis (% wt) of silver in **PenAg** was also confirmed by EDX measurements. EDX chemical map (Figure 1) shows that the relative contents of O and Ag in **PenAg** are 43.53% and 56.47% *w*/*w*. This corresponds to O/Ag molar ratio of 5/1, which is in agreement with the corresponding one of **PenAg** ([Ag(C_16_H_17_N_2_O_4_S)(CH_3_OH)]_2_).

*Vibrational spectroscopy:* The IR spectrum of **PenNa** shows vibration bands at 1621 and at 1418 cm^−1^, which are assigned at v_as_(-COO-) and v_s_(-COO-) of the carboxylic group (Figure S1) [15,16]. These bands are shifted in the case of **PenAg**, at 1611 and 1414 cm^−1^, respectively (Figure S1). Thus the Δ*v* values of **PenNa** and **PenAg** are 204 cm^−1^ and 197 cm^−1^. Monodentate coordination of the carboxylic group results in a significantly higher difference values Δv than those observed for the ionic compounds of the ligand [17], while when the ligand chelates, the Δv is considerably smaller than that observed for its ionic compounds. For asymmetric bidentate coordination, the values are in the range of monodentate one [17]. When the -COO- group bridges metal ions, the Δv values are higher than that of the chelating mode and nearly the same as that observed for ionic compounds [17]. Since the Δv value of **PenAg** is nearly the same with the corresponding one of **PenNa**, a bridging coordination mode of the carboxylic group towards the Ag(I) is concluded [17]. The vibration band of 3370 cm^−1^ in the IR spectrum of **PenAg** corresponds to N–H, which was observed at 3354 cm^−1^ in the case of **PenNa** [15,16]. The vibration band of v(C=O) of the b-lactam at 1777 cm^−1^ is not shifted in the case of **PenAg**. However, the vibration band of the amide carboxyl group is shifted in **PenAg** at 1670 cm^−1^, from 1700 cm^−1^, where it is observed in the case of **PenNa** [15,16]. The IR spectrum of **PenAg** confirms the coordination of **PenNa** to silver(I) ion with a chelating mode.

### 2.3. Solution Studies

**Figure 2:**
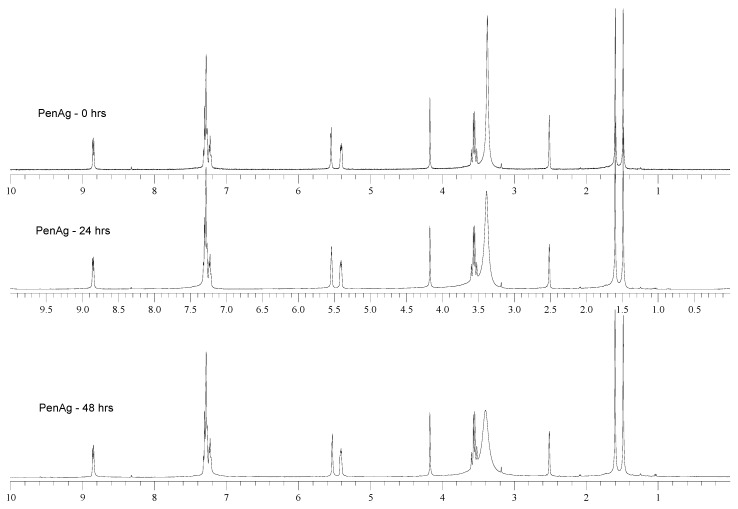
^1^H-NMR spectrum in DMSO-*d_6_* of **PenAg** and the corresponding ones after 0, 24 and 48 h.

*Stability studies:* The stability of **PenAg** was tested in DMSO-*d_6_* solution, by ^1^H-NMR spectroscopy, for periods of 0, 24 and 48 h (Figure 2). No any variations were observed between the initial NMR spectrum and the corresponding ones after 24 and 48 h, suggesting the stability of **PenAg** in solution for 48 h, at least (Figure 2). The period of at least 48 h for the stability of the compound was chosen because the biological experiments require a maximum of 48 h of incubation of the microbes with the compound; DMSO was used for the dilution of **PenAg** in the stock solution (0.01 M) for the biological experiments.

Moreover, the UV–Vis spectrum **PenAg** in dd water is similar to the corresponding one recorded after 24 h, suggesting its stability (Figure S2).

**Figure 3:**
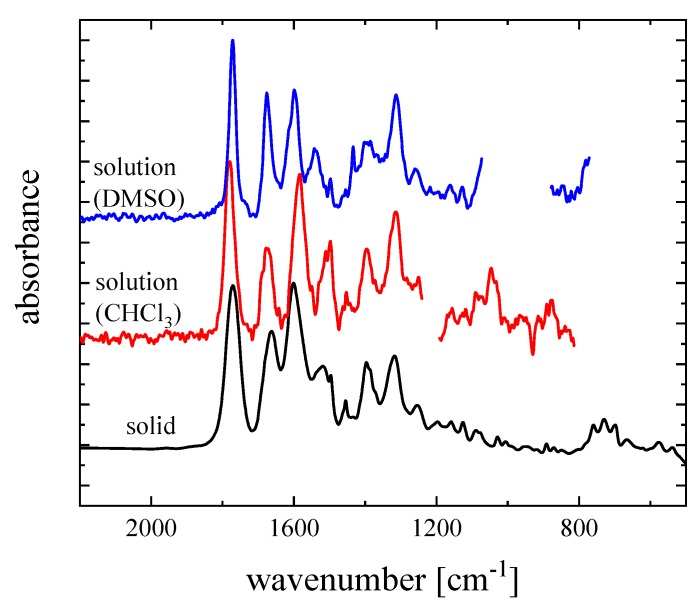
ATR-FTIR spectra of solutions with DMSO and CHCl_3_ as solvents and of the solid phase. The spectra exhibit characteristic similarities in both liquid and solid phases.

*Attenuated total reflection spectroscopy (FT-IR-ATR)*: in order to confirm the retention of the formula of **PenAg** in solutions, FT-IR-ATR spectra were recorded. This is an important tool to find out whether the activity is attributed to synergistic effect of the antibiotic and silver(I) or to the superimposition of the properties, of the ingredients of a mixture. Figure 3 shows the spectrum of the solid **PenAg** obtained with a KBr disc in transmittance mode. The spectra of pure DMSO and CHCl_3_ solvents have been measured and subtracted from the experimental spectra of solutions in order to estimate the excess spectral intensities attributed to the solute. The resulting spectra of the solutions for both solvents are also shown in Figure 3 for comparison. The spectra of the liquid and solid phases reveal characteristic resemblance in the fingerprint region, implying structural similarities. The high-frequency, broad spectral envelope observed in the spectrum of the solid corresponds to water traces originating from KBr. The missing spectral regions correspond to solvent interference regions/residuals after the subtraction procedure.

The Pearson product-moment correlation coefficients (PPMCC) have been utilized to quantitatively follow the linear correlation between the spectra corresponding to the solid and liquid sample presented in Figure 3. The results are presented in Table 1 revealing ≈79% and ≈81% correlations between solid/CHCl_3_-solution and solid/DMSO-solution, respectively.

**Figure 4:**
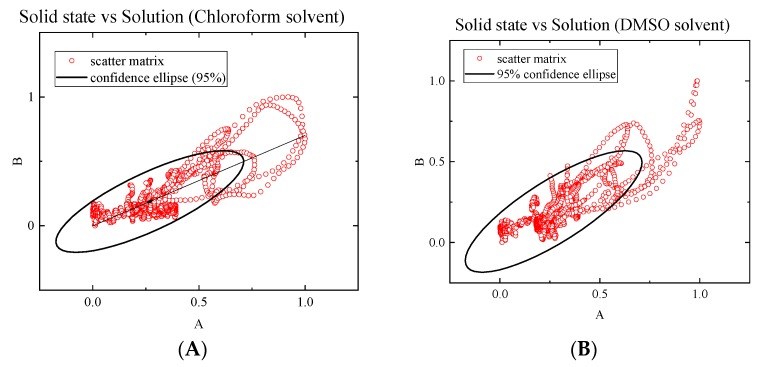
Confidence ellipses calculated from Pearson-type correlations for the solid and the solution with CHCl_3_ (**A**) or DMSO (**B**) used as solvent. See text for details.

The confidence ellipses for both sets, which include points of the scatter matrix with correlations higher than 95%, are shown in Figure 4. Every point of the scatter matrix corresponds to a specific point of the IR spectrum. Most of the points are inside confidence ellipses, indicating spectral similarities. Possible small frequency shifts and bandwidth increases observed in the spectra are due to strong interactions between solvent and solute molecules in solutions.

*Generalized two-dimensional correlation spectroscopy:* In general, the 2D correlation spectroscopy extents the spectral peaks over a second dimension obtaining a 2D correlation map, thereby permitting correlation analysis of the bands in order to investigate the intra and inter-molecular interactions [18]. In order to calculate the dynamic spectra, the first step is to subtract from the from the spectral intensity y(ν,t), the step-averaged reference spectrum y(ν). The second step is the calculation of the Y_1_(ω) and the conjugate transform Y_2_(ω) of the dynamic spectra, where ω corresponds to the Fourier frequency component of the step-dependent signal variation. Thus, the 2D correlation between the y(ν_1_,t) and y(ν_2_,t) is estimated from:

(1) $$X(\nu_{1},\nu_{2})=\Phi (\nu_{1},\nu_{2})+i\cdot \Psi (\nu_{1},\nu_{2}).$$

In the last equation, Χ(ν_1_,ν_2_) and Φ(ν_1_,ν_2_) are the real and imaginary components of Χ(ν_1_,ν_2_) known as synchronous and asynchronous correlation spectra, respectively. The correlation between simultaneous varying spectral intensities is reflected in the synchronous representation. In asynchronous representation, the correlation maximizes when the intensity variations at two wavenumbers are orthogonal, indicating non-comparability of the spectral changes. All calculations described above are relatively complex and were performed by means of a home-made code written in GNU Octave [19]. We will restrict our analysis only in the synchronous 2-D correlation spectra, since no complementary information is obtained from the corresponding asynchronous spectra.

**Figure 5:**
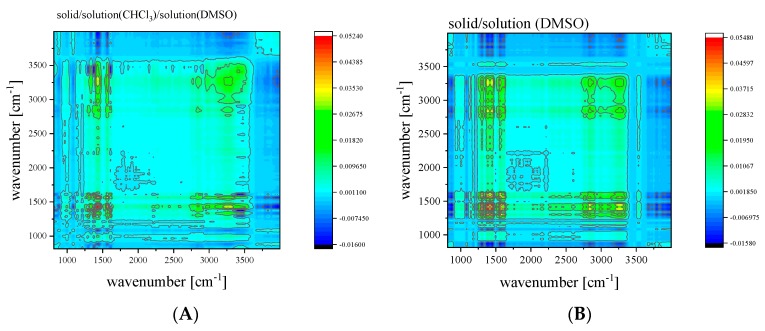
Synchronous 2-D correlation spectra constructed from the vibrational spectra of the solid and CHCl_3_ (**A**) or DMSO (**B**) solution presented in Figure 3.

The purpose of this analysis is to establish the structural similarity between solid phase and solutions utilizing the 2-D correlation methodology. The synchronous correlation maps constructed from the vibrational spectra of solid and CHCl_3_/solution, of solid and DMSO/solution, and for the complete set of spectra, are presented in Figure 5. In all cases, the synchronous 2-D correlation color map reveals simultaneous or coincidental changes. The spectra are fully symmetrical with respect to the diagonal line, indicating almost complete spectral similarity, and thus, structural resemblance. The peaks observed in the diagonal line are called auto-peaks, and are positive, as expected. The observed off-diagonal peaks, known as cross-peaks, represent the synchronicity of the spectral variations between the two spectral coordinates, and their intensities are positive or negative. The cross-peak intensity becomes negative in case where one of the spectral intensities is increasing, while the other one is decreasing. The peak resolution of 2-D infrared correlation spectra is substantially enhanced by spreading the strongly overlapping peaks along the second dimension, allowing a deeper understanding of the structure.

*^1^H NMR studies in DMSO-d_6_ using 400 MHz NMR apparatus:* The ^1^H-NMR spectrum of **PenNa** in DMSO-d_6_ shows a distinct resonance signal for the H[N] (Scheme 1) at 8.73 (br) ppm which is shifted at 8.84 (br) ppm upon coordination of Pen^−^ to Ag(I) in **PenAg** (Figure S3) [20,21]. The resonance signals in the spectrum of **PenNa** at 7.30 and 7.22 (m) ppm, were assigned to the aromatic protons of the benzyl group of penicillin G and they were observed without shift in the case of the spectrum of **PenAg** (7.32 and 7.22 (m) ppm). The signals at 5.35 and 5.34 (m) are attributable to H[C9] and H[C8] respectively (Scheme 1) and they shifted to 5.54 (s) and 5.40 (s) ppm respectively [20,21]. The signal at 3.89 (s) ppm in the spectrum of **PenNa** was assigned to the H[C13], (Scheme 1) but was observed at 4.17 (s) ppm in the corresponding one of **PenAg** [20,21]. The strong downfield shift of this proton confirms the coordination of penicillin on silver(I) ion. The resonance signals in the spectrum of **PenNa** at 3.58 and 3.49 (q) ppm were attributed to the H[C5] protons (Scheme 1), which undergo no shift in **PenAg** (3.59 and 3.52 (q) ppm). This suggests that there is no involvement of the amide carbonyl group in the coordination of **PenNa** towards silver(I). The resonance signal at 3.18 ppm which was observed only in the spectrum of **PenAg**, was attributed to coordinated methanol, suggesting the presences of methanol in the coordination sphere of Ag(I) (Figure S3). The two singlet signals at 1.59 and 1.46 (s) ppm in the spectrum of **PenNa** were assigned to the H[C11,12], but they undergo no shift in **PenAg** (1.59 and 1.48 (s) ppm respectively) (Figure S3).

*Structure of **PenAg** by NMR spectroscopy with 500MHz LC-NMR instrumentation*: An NMR study of **PenNa** has been previously reported [22,23]. Herein the binding mode of **PenNa** at **PenAg** is reported. Two deuterated solvents, DMSO-d_6_ and MeOD-d_4_ were used for the study of both **PenNa** and **PenAg**. In order to study hydrogen bonding of the amide proton in **PenNa**, the temperature coefficient was determined in DMSO-d_6_ solvent. For a range of 293–313 K, the temperature coefficient of the amide proton (H7) was determined to be −8.9 ppb/K. It is generally accepted that temperature coefficients with values higher than −4.5 ppm/K are strongly indicative of intramolecular H-bonds, whereas lower values suggest strong interactions with solvent [23,24,25]. Moreover, Δδ/ΔT values in at about −7.5 ppb/K indicate weakly hydrogen bonded amide protons [26]. Moreover, as recently reported for hydrogen bond motif “C5” geometry [27], the amide proton might participate in such a “C5” geometry favoring intramolecular hydrogen bonding. Nevertheless, the strong interaction of the amide proton with DMSO-d_6_, stronger than in MeOD-d_4_, seems to be the dominating interaction. The amide proton of **PenNa** in DMSO-d_6_ resonates at 8.709 ppm, indicating a hydrogen bonded state, in contrast to MeOD-d_4_, for which the signal is observed at 8.141 ppm (Δδ(DMSO-*d_6_*−MeOD-*d_4_*) = 0.57 ppm). The reported possible intramolecular hydrogen bond behavior could explain the partial and not full deuterium exchange of the amide proton in MeOD-d_4_ solvent (the integral of H7 is 0.3 in MeOD-d_4_ instead of 1.0 in DMSO-d_6_).

**Figure 6:**
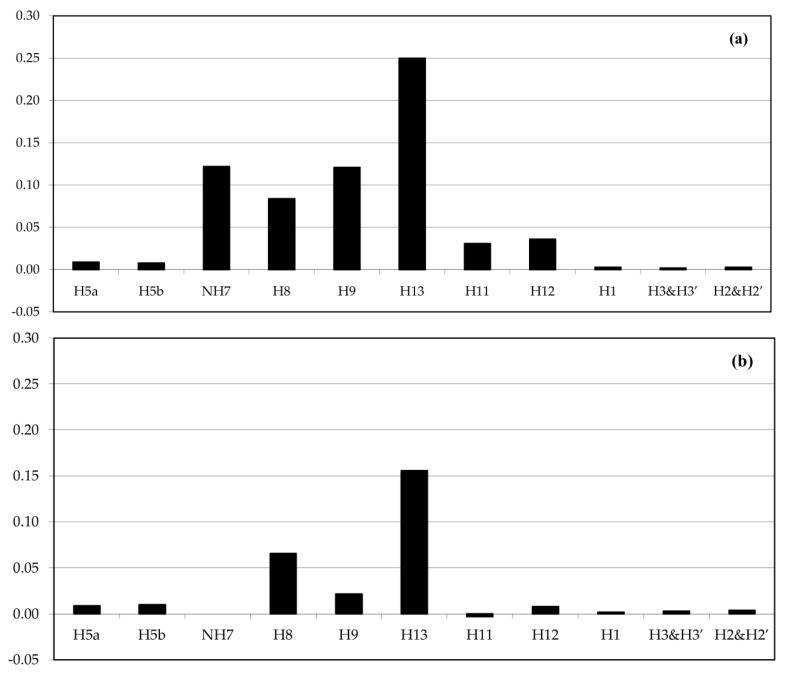
The Δδ for the **PenNa** and the **PenAg** in DMSO-d_6_ (**a**) and in MeOD-d_4_ (**b**).

*Assignment of the **PenAg** NMR spectrum*: Figure 6 shows the Δδ between **PenNa** and **PenAg** in DMSO-d_6_ and in MeOD-d_4_. Since the chemical shifts of the **PenNa** are concentration dependent, similar concentration levels were studied.

The protons that mostly are affected are H13, H9 and H8, with the H13 (Scheme 1) being strongly deshielded, and H9 and H8 (Scheme 1) being less deshielded. In DMSO-d_6_ the deshielding of the above protons is observed and the Δδ values of **PenAg** compared to **PenNa** for the H13, H9 and H8 protons (Scheme 1) are 0.25, 0.12 and 0.08 ppm, respectively. In MeOD-d_4_, the Δδ values of **PenAg** compared to **PenNa** resonances are 0.16, 0.02 and 0.07 ppm, respectively. As shown, H13 proton is strongly deshielded either in MeOD-d_4_ or in DMSO-d_6_, followed by strong deshielding of H9 and H8 protons in DMSO-d_6_, whereas the opposite is true in MeOD-d_4_. The H12 and H11 protons are less deshielded and the H5 protons are also affected, whereas the protons of the aromatic ring are not. Following these data, a stronger deshielding is observed when DMSO-d_6_ is used, probably due to the type of complexation. As shown in Figure 6, methanol molecules also coordinate to Ag(I), upon its coordination resulting the less intense deshielding effect.

An important difference when the two solvents are used for the current study, is that the H7 amide proton is strongly deshielded (Δδ[(PenNa-PenAg] = 0.12 ppm) in DMSO-d_6_, whereas in MeOD-d_4_ the H7 amide proton is not observed for the **PenAg**. Therefore, there is a crucial difference for the **PenAg** when it is studied in two different solvents.

In a hydrogen bonding accepting solvent, such as dimethylsulfoxide, a strong interaction of the solvent with the amide proton exists, and hydrogen bonds are present in the structure of the molecule. The coordination of Ag(I) to the carboxyl moiety of penicillin results in the deshielding the H13 proton. Moreover, it seems that Ag(I) also interacts with sulfur in the thietane ring, thereby affecting (deshielding) H9 and H8 protons. Since a stronger hydrogen amide bond is observed on the **PenAg** complex, the intramolecular ‘C5′ type hydrogen bond could be more favored, probably due to conformational/structural changes, most likely on an angle between the N–H proton and C=O group upon the coordination of Ag(I) in the molecule.

On the contrary, when MeOD-d_4_ was used for the study of the **PenAg**, a similar behavior for H13 is observed, indicating the coordination of the Ag(I) to the carboxylic moiety. Furthermore, H7 amide proton was not observed due to deuterium exchange of the amide proton in MeOD-d_4_ solvent. Following the above, a shifting (deshielding) of the H8 (Δδ = 0.07) and H9 protons (Δδ = 0.02 ppm) is observed. It should be noted that broad peaks are observed for the H8 and H9 protons (Δv_1/2_ ≈ 15 and 10 Hz, respectively), due to the rapidly isomerizing of the structure. Moreover, after the exchange of the H7 amide proton by deuterium in MeOD-d_4_, the H8 proton is more deshielded compared to the H9 proton. Additionally, since the Δδ of the H9 proton is 0.02 ppm, no coordination of Ag(I) to sulfur atom at the thietane ring could be concluded. The absence of the H7 proton amide proton due the deuterium exchange is confirmed, since in **PenNa**, ≈0.7 presentence of the molecule does not exchange with the deuterium present in MeOD-d_4_.

*Assignment of the **PenNa** NMR spectrum*: It should be noted that a specific geometry appearing on the DMSO-d_6_ solvent is observable. More specificly, H8 proton resonates at 5.331 ppm and its multiplicity is dd with J values of 9.8 and 4.0 Hz. This is due to the anti-coupling to H7 (it is in the range of 8 to 10 Hz) and to the gauss-coupling to H9 (expected less than 5 Hz). On the contrary, in the MeOD-d_4_ solvent, one broad peak for the H8 and H9 protons is seen due to the fast time scale of the phenomenon. ^1^H-^13^C HSQC experimentation reveals the two resonances for the H9 and H8 protons. Moreover, upon the NMR study of the **PenAg** in DMSO-d_6_, the H12 proton is more affected, possibly due to its proximity to the carboxylic carbon, which is in close proximity to the metal coordination.

*The concentration dependence of the **PenNa** chemical shifts using DMSO-d_6_ solvent:* The concentration dependence of the geminal proton resonances in D_2_O has been previously reported [22]. Herein we report our study for the dimethylsulfoxide solvent. For the **PenNa** concentration levels of 1.0, 2.0, and 4.0 mg mL^−1^ in DMSO-d_6_, a linear dependence of the chemical shifts for the H8, H9, H11, H12, and H7 protons was observed, whereas a plateau was reached for H13. The Δδ values for 4.0 and 2.0 mg mL^−1^ were shifted downfield: 0.022 for H7, 0.015 for H9, 0.014 for H8, and 0.007 for H11 and H12 protons. On the contrary, no concentration dependence was observed for the aromatic and the H5 protons. Earlier reports [28] have shown that changes in chemical shift upon concentration variation occur due to aromatic solvent induced shifts, or due to simple dimerization or conventional π–π stacking of solute molecules or molecular aggregation, leading to differently packed supramolecular assemblies or due to formation of intermolecular associations [29].

In order to study the phenomenon, DOSY spectroscopy was used. Diffusion values were measured for the above concentration levels—3.27 × 10^−10^, 2.96 × 10^−10^_,_ and 2.26 × 10^−10^ m^2^ s^−1^, respectively. Thus, upon increase of the **PenNa** concentration, bigger clusters are created, leading to smaller diffusion values. Since the diffusion value depends on the hydrodynamic radius of the molecule (1/r^6^), the increase of the concentration results in the formation of (micellar) solutions, probably through intermolecular associations.

In conclusion, based the NMR study of the **PenNa** and **PenAg** in two selected solvents, between a hydrogen bonding accepting solvent (DMSO-d_6_) and a hydrogen bond solvent (MeOD-d_4_), crucial differences are revealed. Firstly, Ag(I) coordinates with **PenNa** through the carboxylic group. There is a crucial difference for the **PenAg** when studied in the two selected solvents. In the hydrogen bonding accepting solvent, a strong interaction of solvent to amide proton exists and hydrogen bonds are present in the structure of the molecule. On the contrary, when MeOD-d_4_ is used, the H7 amide proton is not observed, probably due to its exchange by deuterium. Secondly, there is a concentration dependence of the chemical shift to the concentration level due to several reasons, such as molecular aggregation or formation of intermolecular associations. The results were also supported by the DOSY spectroscopy of the bigger cluster being present upon increase of the **PenNa** concentration.

*Atomic absorption (AA):* In order to confirm the formula of **PenAg,** graphite furnace atomic absorption spectroscopy (GFAAS) was employed. The mass of the silver is 22.67% *w*/*w*, which is in agreement with the calculated one of [Ag(pen)(CH_3_OH)]_2_ (22.79% *w*/*w*).

**Figure 7:**
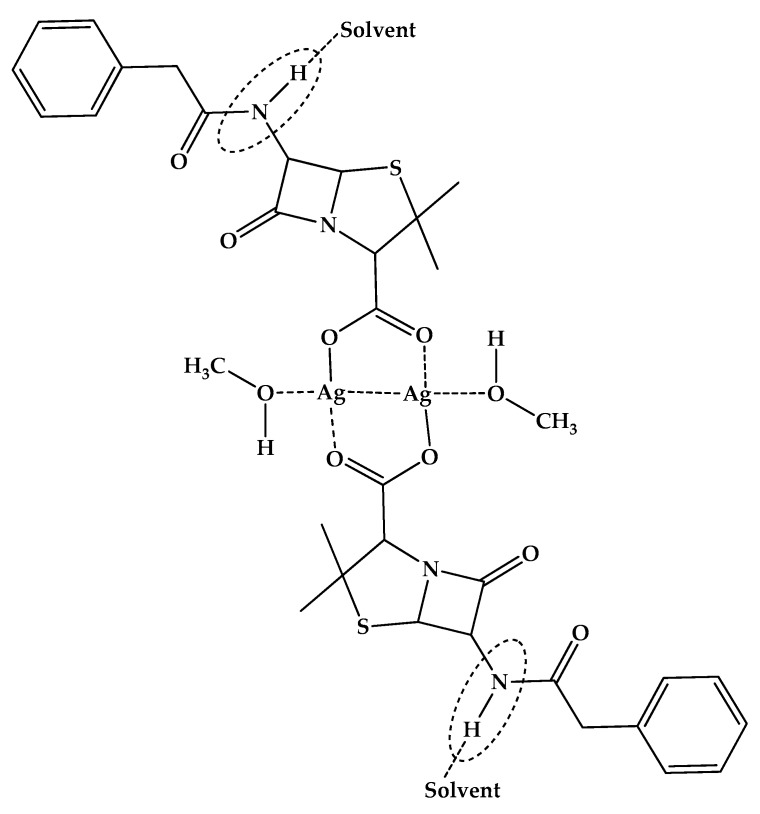
Structure of **PenAg** refined by the analytical and spectroscopic data.

*Proposed structure of **PenAg***: The following structure of **PenAg** (Figure 7) is proposed based on the elemental analysis, XRF, SEM-EDX, FT-IR, ^1^H NMR spectroscopy, HRMS, and AA. The strength of the hydrogen bond H[N]· ··O[solvent] with a hydrogen bonding accepting solvent (DMSO) and a hydrogen bond solvent (MeOD-d_4_) affect the stability of the supramolecular architecture.

### 2.4. Antibacterial Studies

Table 2 compares the MIC, MBC, and IZ of **PenAg**, **PenNa** with the corresponding ones of other CoMeD’s derived from silver(I) or zinc(II) ions with ciprofloxacin (an antibiotic), salicylic acid or citric acid (natural products), and 2-mercapto-nicotinic acid (an antimetabolite) against *P. aeruginosa*, *S. epidermidis* and *S. aureus*.

*The effects of **PenAg** and **PenNa** on the growth of microbial strains:* The antimicrobial potencies of **PenAg** and **PenNa** were tested against Gram negative (*P. aeruginosa*) and Gram positive (*S. epidermidis* and *S. aureus*) bacterial strains by the means of MIC. MIC is defined as the lowest concentration needed for the inhibition of the bacterial growth [30]. The MIC value of **PenAg** towards the Gram negative, *P. aeruginosa* bacteria is 23.00 ± 2.29 μΜ, in contrast to the corresponding one of **PenNa,** which is higher than 2000 μΜ (Table 2). The MIC values of **PenAg** and **PenNa**, on the other hand, against *S. epidermidis,* are 2.41 ± 0.88 and 3.76 ± 1.14 μΜ, respectively, and against *S. aureus* are 0.08 ± 0.02 and 0.14 ± 0.02 μΜ, respectively (Table 2). Thus, **PenAg** shows superior antibacterial effects over **PenNa** towards the Gram positive bacteria (*S. epidermidis* and *S. aureus*) and expanded activity into Gram negative microbes, such as *P. aeruginosa,* where **PenNa** is inactive.

*Evaluation of the minimum bactericidal concentration (MBC):* The MBC is defined as the lowest concentration of an antibacterial agent that can eliminate the 99.9% of the bacterial inoculum [31]. The MBC value of **PenAg** towards *P. aeruginosa* is 46.6 μΜ, (Figure S4). On the contrary, no MBC value against *P. aeruginosa* could be determined for **PenNa** when it was tested at concentrations up to 2000 μΜ, following the general inactivity of penicillins against Gram negative microbes.

**Figure 8:**
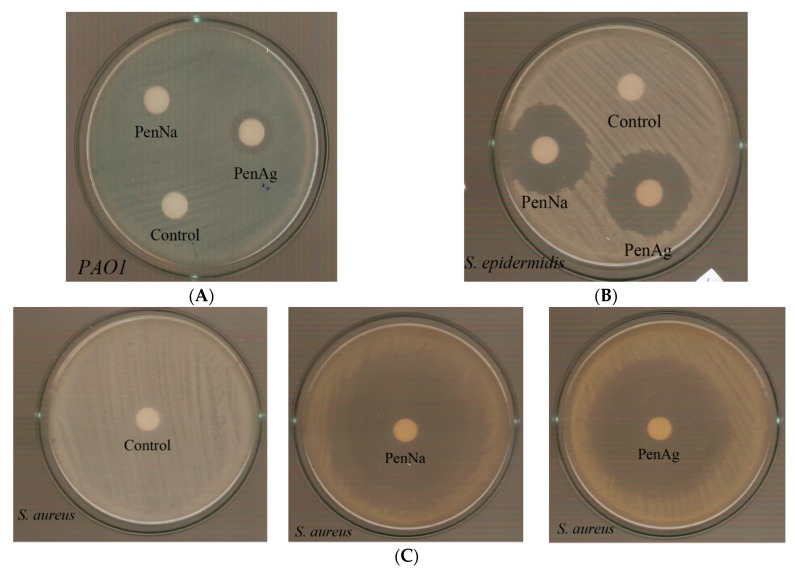
Inhibition zones which are grown in *Pseudomonas aeruginosa* (**A**), *Staphylococcus epidermidis* (**B**), and *Staphylococcus aureus* (**C**) for **PenAg** and **PenNa**.

*Determination of the inhibition zone (IZ) through the agar disk-diffusion method*: The agar disk-diffusion method was used in order to survey the sensitivity of the microorganism to **PenAg** [32]. The diameter of inhibition zones of bacterial growth which were caused by **PenAg** or **PenNa** at the dose of 1 mg/mL against *P. aeruginosa, S. epidermidis, and S. aureus,* were measured after 20 h (Table 2, Figure 8). Although both **PenAg** and **PenNa** inhibit the growth of the Gram positive bacteria equally (*S. epidermidis and S. aureus,* Table 2), this is not the case with the Gram negative one (*P. aeruginosa*), for which the activity of **PenAg** is superior to that of **PenNa**. This confirms the conclusion withdrawn above (see MIC) that the conjugation of silver ions with penicillin expands the activity of **PenNa** to Gram negative microbes such as *P. aeruginosa,* against which the free penicillin is inactive. Moreover this conjugation of silver with penicillin enhances its activity against Gram negative microbes, such as *S. epidermidis and S. aureus.*

Among CoMeD’s derived from the conjugation of silver(I) or zinc(II) ions with biologically active molecules, such as ciprofloxacin (an antibiotic), salicylic acid, or citric acid (natural products), CIPAG exhibits the highest activity against both positive and negative bacteria, with a range of MIC values between 0.46 and 0.61 μΜ (Table 2) and IZ values of 28–34 mm. However, **PenAg** shows the highest antibacterial activity against *S. aureus*, an MIC value of 0.08 ± 0.02 μΜ, and a IZ of 57 mm. Therefore, it is concluded that the conjugation of metal ions with drugs make bacteria more susceptible to antibiotic or agents, while it creates new chemotherapeutics for drug-resistant infections.

### 2.5. In Vivo Toxicity Evaluation, by Brine Shrimp Artemia Salina

The in vivo toxicity of the **PenAg** has been performed using brine shrimp *Artemia salina* assay. The brine shrimp *Artemia salina* lethality test is a preliminary toxicity test, useful for predicting biological activities such as cytotoxic, phototoxic, and pesticidal activities [33]. Thus, pharmacological activities of bioactive compounds can be evaluated with a brine shrimp lethality bioassay [34]. This is due to its widespread distribution, short life cycle, non-selective grazing, and sensitivity to toxic substances [35,36].

The survival (%) of *Artemia salina* larvae in increasing concentrations of solutions with or without **PenAg** after 24 h are summarized in Table S1. The lethality was noted in terms of deaths of larvae. No mortality rate of *brine shrimp* larvae was found upon their incubation with **PenAg** in concentrations up to 220 μM (Table S1). This lethal concentration of **PenAg** is 10, 100 and 2750-fold higher than its MIC values against *P. aeruginosa*, *S. epidermidis,* or *S. aureus* respectively, suggesting a wide therapeutic window and thereby low toxicity.

## 3. Conclusions

Penicillins, especially penicillin G, are among the first line of antimicrobial agents currently in use for the treatment of intra-hospital infections [2]. However penicillins suffer from inactivity against Gram negative bacteria [1]. In order to overcome this disadvantage, the conjugation of penicillin with silver(I) ions was performed. State of the art, analytical, and spectroscopic techniques revealed the CoMeD formula [Ag(pen)(CH_3_OH)]_n_ (**PenAg**). NMR studies reveal that silver(I) coordinates with **PenH** through the carboxylic group.

The antibacterial studies show that **PenAg** is active against Gram negative (*P. aeruginosa*), contrary to penicillin itself, which is inactive. Silver(I) ions, on the other hand, show bacteriocidal activity against Gram negative (*P. aeruginosa*) [8,9]. Moreover, **PenAg** exhibits superior antibacterial activity to the free antibiotic against the Gram positive bacteria (*S. epidermidis* and *S. aureus*). Thus, a synergy in silver(I) and penicillin G properties is achieved by their conjugation within **PenAg**. This is in agreement with the observation of Morones-Ramirez et.al [37] that silver(I) ions exhibit an antimicrobial synergistic effect against Gram negative bacteria, when used in combination with beta-lactams, aminoglycosides, and quinolones [37]. This effect is attributed to the permeabilization of the outer membranes of Gram negative bacteria by silver(I) ions, suspending the existing drugs neutralization. The lethal concentration which is exhibited by **PenAg**, on the other hand, is multiple times its MIC values (up to 2750-fold higher) against all microbial strains used, making **PenAg** a possible candidate towards the development of a new antibiotic with a wide spectrum of activity, giving a new life to penicillin.

## 4. Materials and Methods

*Materials and instruments:* All solvents used were of reagent grade. Tryptone and soy peptone were purchased from Biolife. Agar was purchased from Sigma-Aldrich. Sodium chloride, D(+)-glucose, and di-potassium hydrogen phosphate trihydrate were purchased from Merck. Melting points were measured in open tubes with a Stuart Scientific apparatus and are uncorrected. FT-IR spectra in the region of 4000–370 cm^−1^ were obtained from KBr discs, with a Perkin–Elmer Spectrum GX FT-IR spectrophotometer. The ^1^H-NMR spectra were recorded on a Bruker AC 400 MHz FT-NMR instrument in DMSO-d*_6_* solution. A UV-1600 PC series spectrophotometer of VWR was used to obtain electronic absorption spectra. XRF measurements were carried out using an Am-241 radio isotopic source (exciting radiation 59.5 keV). Brine shrimp eggs (*Artemia salina*) were purchased from Ocean Nutrition.

*Synthesis of [Ag(Pen)(CH_3_OH)]_n_****PenAg****:* A dd water solution of silver nitrate (0.5 mmol, 0.085 g) was added to a methanolic solution of benzyl-penicillin sodium (PenNa) (0.5 mmol, 0.178 g) and the resulting suspension was stirred for 5 min. The white precipitation was filtered off and it was successively washed with 4 mL of cold methanol/water solution (1:1) and 2 mL of acetone.

**PenAg**: Yield 67%; melting point: 124–127 °C. Elemental analysis found: C: 43.64%; H: 4.26%; N: 5.70%; S: 6.93%; Ag: (21.35 ± 2.33)% Calculated for C_34_H_42_Ag_2_O_10_N_4_S_2_, C: 43.15%; H: 4.47%; N: 5.92%; S: 6.77%; Ag: 22.79%;. IR (cm^−1^), (KBr): 2964 m, 1774 vs, 1752 m, 1670 s, 1612 vs, 1492 s, 1396 s, 1384 s, 1335 s, 1316 m, 1249 m, 1211 w, 1098 w, 760 w, 719 m, 698 w, 664 w; ^1^H NMR (ppm) in DMSO-d*_6_*: 8.84 (br, H[N]), 7.32–7.22 (m, protons of the benzyl group of penicillin G), 5.54 (s, H[C4^a^]), 5.40 (s, H[C5]), 3.89 (s H[C3]), 3.59–3.52 (q, H[PhC]), 1.59–1.48 (s, H[Me^a,b^]), UV–Vis (MeOH): λ = 213 nm (log*ε* = 4.02)

*Elemental analysis of silver:* The content of **PenAg** in silver was determined as silver chloride. A known quantity of **PenAg** was dissolved in 1 mL of extra pure nitric acid. The resulting solution was heated to 150 °C in oil bath for 2 h. The clear solution was then diluted to 15 mL using dd water, followed by addition of 5 mL saturated solution of NaCl. The white precipitation of AgCl was filtered off and dried in an oven overnight. The dry solid was weighted to give the silver content of **PenAg**.

*NMR analysis:* The solution was transferred into a 5 mm NMR tube. NMR experiments were performed on a Bruker AV500 spectrometer (Bruker Biospin, Rheinstetten, Germany) at 298 K using the Topsin 2.1 suite. All 1D ^1^H NMR spectra were collected using a 30° flip angle, a spectral width of 14 ppm with the same receiver gain value, a relaxation delay of 5 s, a number of transients of 512, and an acquisition time of 4.3 s. Sixty-four thousand data points were collected and the free induction decay (FIDs) were treated using a line broadening exponential function of 0.3 Hz. Phase adjustment and baseline correction was carried out using Topspin 2.1 suite. Signal integration was manually performed.

2D ^1^H-^1^H TOCSY, ^1^H-^13^C HSQC, and ^1^H-^13^C HMBC NMR experiments were recorded using standard Bruker software. DOSY experiments were performed using the bipolar pulse longitudinal eddy current delay (BPPLED). For a specific diffusion time (∆) the magnetic field pulse gradient (δ) was optimized in order to obtain a 1%–5% residual signal with the maximum gradient strength. Typically, 100 and 3.0 ms were used for ∆ and δ, respectively. 16 BPPLED spectra on 16 K data points were collected and the eddy current delay (T_e_) was set to 5 ms. The pulse gradient was increased from 2% to 95% of the maximum gradient strength using a linear ramp. The temperature was set to 298 K with an air flow of 615 L h^−1^ in order to minimize convection effects and to maintain temperature stability due to sample heating during the magnetic field gradient. After Fourier transformation and baseline correction, the diffusion dimension was treated with the Topspin 2.1 suite.

*NMR assignment:*

**PenNa:**^1^H-NMR (in DMSO-d_6_, 500.13 MHz)

δ = 1.469 (s, 3H, H11), 1.593 (s, 3H, H12), 3.590 (d, ^2^*J* = 13.95 Hz, 1H, H5a), 3.541 (d, ^2^*J* = 13.95 Hz, 1H, H5b), 3.872 (s, 1H, H13), 5.331 (dd, *J* = 7.1, 3.9 Hz, 1H, H8), 5.339 (d, ^3^*J* = 3.9 Hz, 1H, H9), 8.709 (d, ^3^*J* = 7.1 Hz, 1H, H7), 7.250 (t, *J* = 7.0 Hz, 1H, H1), 7.293 (d, *J* = 7.0 Hz, 2H, H3&H3′), 7.327 (t, *J* = 7.0 Hz, 2H, H2, and H2′).

**PenNa:**^1^H-NMR (in MeOD-d_4_, 500.13 MHz)

δ = 1.577 (s, 3H, H11), 1.658 (s, 3H, H12) 3.597 (d, ^2^*J* = 14.50 Hz, 1H, H5a), 3.646 (d, ^2^*J* = 14.50 Hz, 1H, H5b), 4.208 (s, 1H, H13), 5.498 (b, 1H, H8), 5.492 (b, 1H, H9), 8.141 (s, 0.2H, H7), 7.272 (m, 1H, H1), 7.327 (m, 2H, H3 and H3′), 7.334 (m, 2H, H2 and H2′).

**PenNa:**^1^H-^13^C NMR (in MeOD-d_4_, 500.13 MHz)

Δ = 27.88 (C11), 31.89 (C12), 43.41 (C5), 75.07 (C13), 69.24 (C**8**), 58.55 (C**9**), 128.00 (C1), 130.51 (C3), 131.38 (C2).

**PenAg:**^1^H-NMR (in DMSO-d_6_, 500.13 MHz)

δ = 1.500 (s, 3H, H11), 1.629 (s, 3H, H12) 3.599 (d, ^2^*J* = 13.95 Hz, 1H, H5a), 3.549 (d, ^2^*J* = 13.95 Hz, 1H, H5b), 4.122 (s, 1H, H13), 5.415 (dd, *J* = 7.6, 3.9 Hz, 1H, H8), 5.460 (d, ^3^*J* = 3.9 Hz, 1H, H9), 8.831 (d, ^3^*J* = 7.6 Hz, 1H, H7), 7.253 (t, *J* = 7.1 Hz, 1H, H1), 7.295 (d, *J* = 6.7 Hz, 2H, H3 and H3′), 7.330 (t, *J* = 7.2 Hz, 2H, H2 and H2′).

**PenAg:**^1^H-NMR (in MeOD-d_4_, 500.13 MHz)

δ = 1.574 (s, 3H, H11), 1.666 (s, 3H, H12), 3.607 (d, ^2^*J* = 14.50 Hz, 1H, H5a), 3.655 (d, ^2^*J*= 14.50 Hz, 1H, H5b), 4.364 (s, 1H, H13), 5.520 (dd, *J*= 7.1, 3.9 Hz, 1H, H8), 5.558 (d, ^3^*J*= 3.9 Hz, 1H, H9), 7.274 (m, 1H, H1), 7.330 (m, 2H, H3 and H3′), 7.338 (m, 2H, H2 and H2′).

*Atomic absorption:* Solutions of known amounts of **PenAg** were dissolved in 500 μL ultra pure nitric acid. The detection of silver mass in the sample was performed with a graphite furnace atomic absorption spectrophotometer (GFAAS).

*ATR-FTIR:* A mid-IR FTIR spectrometer by Bruker (Alpha model) equipped with an ATR accessory (ZnSe crystal, single reflection, incident angle 45°) was used to collect the absorption spectra of liquids at room temperature and pressure bearing a DTGS detector. In order to obtain a sufficient signal-to-noise, 32 scans with a nominal resolution of 2 cm^−1^ were averaged. After each measurement, the crystal surface was carefully cleaned with spectroscopic grade isopropanol. The Fourier-transform infrared (FTIR) spectra of solids were measured in KBr pellets in transmittance mode under same spectral conditions. A background spectrum was recorded prior to any FTIR and ATR measurement using the atmospheric vapor correction. All spectra have been vector normalized for comparison reasons.

*Bacterial strains:* For the antibacterial experiments the strain of *Staphylococcus epidermidis* (ATCC^®^ 14990™), *S. aureus* subsp. *aureus* (ATCC^®^ 25923™), and *P. aeruginosa PAO1* were used.

*Solvents used:* The biological experiments were performed in DMSO/broth medium (up to 0.0089% *v*/*v* DMSO in broth). A stock solution of **PenAg** (0.01 M) in DMSO was freshly prepared and diluted with microbe’s culture medium to the desired concentration.

*The effects of **PenAg** or **PenNa** on the growth of microbial strains*: This study was performed according standard procedure, which is also described elsewhere [8,9,10,11,12,30]. Briefly, the bacterial strains were streaked onto in trypticase soy agar. The plates were incubated for 18–24 h at 37 °C. Three to five isolated colonies were selected of the same morphological appearance from the fresh agar plate using a sterile loop and transfer into a tube containing 2 mL of sterile saline solution. The optical density at 620 nm is 0.1, which corresponds in to 10^8^ cfu/mL. The final inoculum size for broth dilution is 5 × 10^5^ cfu/mL. The total volume of the culture solution treated by **PenAg** or **PenNa**, and the total volumes of the positive and negative controls, were all 2 mL. The range of concentrations of **PenAg** or **PenNa** was 0.004–2000 μΜ. The growth was assessed after incubation for 20 h. The minimal inhibitory concentration (MIC) was determined as the concentration of the compound which inhibits the visible growth of the bacterium being investigated. The optical density of the solution at 620 nm compared to the concentrations graph led to the MIC determination [8,9,10,11,12,30].

*Minimum bactericidal concentration testing:* This study was performed according standard procedure which is also described elsewhere [8,9,10,11,12,31]. Bacteria were initially cultivated in the presence of **PenAg** in broth culture for 20 h. The MBC values were determined in duplicate, by subculturing 4 μL of the broth an agar plate [8,9,10,11,12,31]. Colony growth suggests non bactericidal activity of a metallodrug. Thus, minimum bactericidal concentration was then defined as the lowest concentration at which a tested compound inhibited the microbe’s growth completely [8,9,10,11,12,31].

*Determination of the inhibition zone (IZ) through the agar disk-diffusion method:* This study was performed according to standard procedure, which is also described elsewhere [8,9,10,11,12,32]. Thus, agar plates were inoculated with a standardized inoculum (10^8^ cfu/mL) of the tested microorganism. Filter paper disks (9 mm in diameter), which had been previously soaked by **PenAg** or **PenNa** (1 mg/mL), were placed on the agar surface. The Petri dishes were incubated for 20 h, and then the diameters of the inhibition zones were measured [8,9,10,11,12,32].

*Evaluation of toxicity with brine shrimp assay*: Brine shrimp assay was performed by a method previously described [38]. One gram cysts were initially hydrated in freshwater for one hour in a separating funnel or cone shaped container. Seawater was prepared by dissolving 17 g of sea salt in 500 mL of distilled water [38,39]. The cone was facilitated with good aeration for 48 h at room temperature and under continuous illumination. After hatching, nauplii released from the egg shells were collected at the bright side of the cone (near the light source) by using micropipette. The larvae were isolated from the eggs by aliquoting them in small beaker containing NaCl 0.9% [40]. An aliquot (0.1 mL) containing about 48 to 58 nauplii was introduced to each well of 24-well plate, and **PenAg** were added in each well. The final volume of each well was 1 mL with NaCl 0.9%. The brine shrimps were observed after 24 h, using a stereoscope. Larvae were considered dead if they did not exhibit any internal or external movement in 10 s of observation. Each experiment was repeated three times.

## Figures and Tables

**Table 1:** Pearson correlation coefficients for solid and solution with CHCl_3_ or DMSO used as solvents.

	Solid/KBr	CHCl_3_			Solid/KBr	DMSO
**Solid/KBr**	1	0.78715		**Solid/KBr**	1	0.80955
**CH_3_Cl**	0.78715	1		**DMSO**	0.80955	1

**Table 2:** Minimum inhibitory concentration (MIC), minimum bactericidal concentration (MBC), and inhibition zones (IZ) of **PenAg** and **PenNa** in comparison with the corresponding ones of other silver and zinc complexes tested against *P. aeruginosa*, *S. epidermidis,* and *S. aureus*.

Compound	MIC values (μΜ)			Ref.
	*P. aeruginosa*	*S. epidermidis*	*S. aureus*	
**PenAg**	23.00 ± 2.29	2.41 ± 0.88	0.08 ± 0.02	[*]
**PenNa**	>2000	3.76 ± 1.14	0.14 ± 0.02	[*]
CIPAG	0.61 ± 0.14	0.46 ± 0.08	0.54 ± 0.07	[8]
([CIPH_2_^+^]·Cl^−^)	1.17 ± 0.22	1.08 ± 0.12	1.45 ± 0.12	[8]
AGSAL-1	28	50	42	[9]
salH_2_	>1250	>1250	>1250	[9]
AGMNA	25.7 ± 2.4	-	42.0 ± 0.3	[10]
MNAH_2_	>300	-	>300	[10]
[Zn_3_(CitH)_2_]	>250	119.2 ± 18.1	183.3 ± 31.4	[12]
CitH_4_	>250	>250	>250	[12]
	MBC values (μΜ)			
**PenAg**	46.6	-	-	[*]
**PenNa**	2000	-	-	[*]
CIPAG	0.7	0.8	1.0	[8]
([CIPH_2_^+^]·Cl^−^)	1.60	1.60	2.00	[8]
AGSAL-1	33.0	90.0	50.0	[9]
salH_2_	ND	ND	ND	[9]
AGMNA	61.1	-	85.7	[10]
MNAH_2_	153.3	-	135.0	[10]
[Zn_3_(CitH)_2_]	>250	201.7	>250	[12]
CitH_4_	>250	>250	>250	[12]
	Inhibition zone (mm)			
**Control/DMSO**	10	10	10	
**PenAg**	17	34	57	[*]
**PenNa**	10	34	60	[*]
CIPAG	32	34	28	[8]
([CIPH_2_^+^]·Cl^−^)	30	36	24	[8]
AGSAL-1	13	13	12	[9]
salH_2_	10	10	10	[9]
AGMNA	21.8	-	22.1	[10]
MNAH_2_	10	10	10	[10]
[Zn_3_(CitH)_2_]	10	10	10	[12]
CitH_4_	10	10	10	[12]

[*] this work, ND = not determined, CIPAG= {[Ag(CIPH)_2_]NO_3_∙0.75MeOH∙1.2H_2_O}, CIPH = ciprofloxacin, [CIPH_2_^+^]·Cl^−^ = hydrochloric salt of ciprofloxacin, AGSAL-1 = [Ag(salH)]_2_, salH_2_ = salicylic acid, AGMNA = {[Ag_6_(μ_3_-Hmna)_4_(μ_3_-mna)_2_]^2^^−^·[(Et_3_NH)^+^]_2_·(DMSO)_2_·(H_2_O)}, MNAH_2_ = 2-mercapto-nicotinic acid, CitH_4_ = citric acid.

## Supplementary Materials

The following are available online at https://www.mdpi.com/2079-6382/9/1/25/s1. Table S1: % Survival of *Artemia salina* larvae in increasing concentrations of solutions with or without **PenAg** or **PenNa** after 24 h. Figure S1: FT-IR spectra of **PenAg** and **PenNa**. Figure S2: UV–Vis spectra of **PenAg** at 0 and 24 h. **Figure S3**: ^1^H-NMR spectra of **PenAg** (upper) and **PenNa** (down) in DMSO-d*_6_*. Figure S4: Minimum bactericidal concentration of **PenAg** against *P. aeruginosa* (*PAO1*).
