# Supplementary material for: Conjugation of Penicillin-G with Silver(I) Ions Expands Its Antimicrobial Activity against Gram Negative Bacteria
Source: Antibiotics (Basel). 2020 Jan 13;9(1):25. doi: 10.3390/antibiotics9010025 (PMC7168214; doi:10.3390/antibiotics9010025)
Supplement: Supplementary file 1 [file antibiotics-09-00025-s001.pdf]

antibiotics

## Supplementary Information

# Conjugation of penicillin-G with silver(I) ions expands its antimicrobial activity, against Gram negative bacteria.

**I. Ketikidis[1], C.N. Banti[1,*], N. Kourkoumelis[2], C.G. Tsiafoulis[3,*], C. Papachristodoulou[4], A.G. Kalampounias[5], S.K. Hadjikakou[1,6,*]**

[1] Inorganic Chemistry laboratory, Department of Chemistry, University of Ioannina, 45110, Ioannina, Greece; giannhsketikidis@yahoo.gr, cbanti@uoi.gr, shadjika@uoi.gr

[2] Medical Physics Laboratory, Medical School, University of Ioannina, Greece; nkourkou@uoi.gr

[3] Laboratory of Analytical Chemistry, Department of Chemistry University of Ioannina, Greece; ctsiafou@uoi.gr

[4] Department of Physics, University of Ioannina, Ioannina, Greece; xpapaxri@uoi.gr

[5] Physical Chemistry Laboratory, Department of Chemistry, University of Ioannina, 45110 Ioannina, Greece; akalamp@uoi.gr

[6] University Research Center of Ioannina (URCI), Institute of Materials Science and Computing, Ioannina, Greece; shadjika@uoi.gr

* Correspondence: cbanti@uoi.gr (CBN), ctsiafou@uoi.gr (CGT), shadjika@uoi.gr (SKH); Tel.: +30-26510-08374 (SKH)

**Table S1.** % Survival of *Artemia salina* larvae in increasing concentrations of solutions with or without **PenAg** or **PenNa** after 24 hrs

| **PenAg** | |
|---|---|
| **Concentration (μM)** | **% Survival** |
| 37.0 | 94.7±2.5 |
| 74.5 | 87.3±5.0 |
| 150.0 | 82.6±4.7 |
| 220.0 | 63.4±6.1 |
| 1050 | 11.0±5.0 |

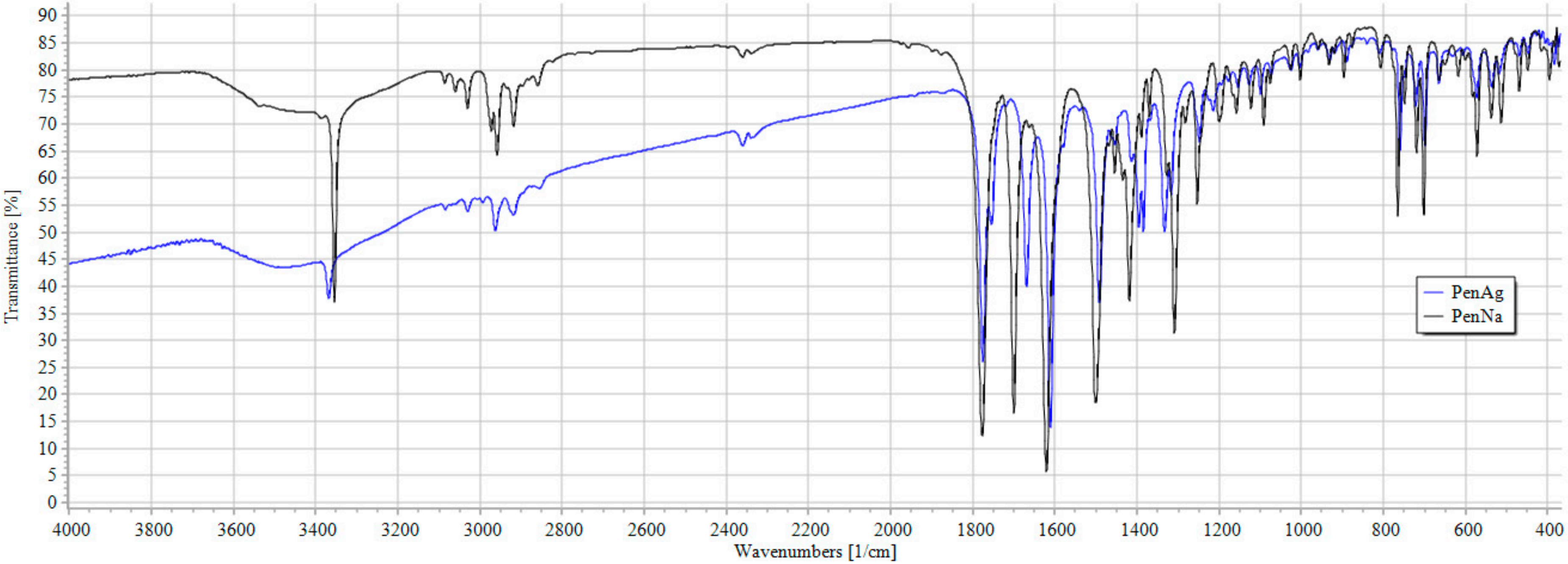


**Figure S1**. FT-IR spectra of **PenAg** and **PenNa** .

antibiotics

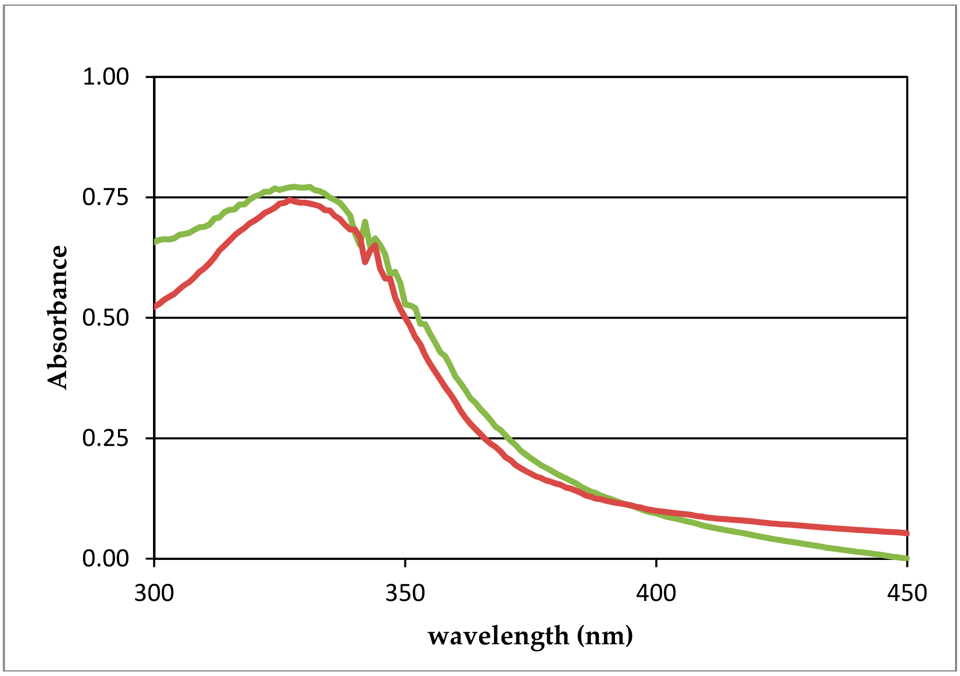


**Figure S2.** Uv-Vis spectrum of **PenAg** in dd water (5 $10^{-4}$ M) (red) and the corresponding one after 24 hrs (Green).

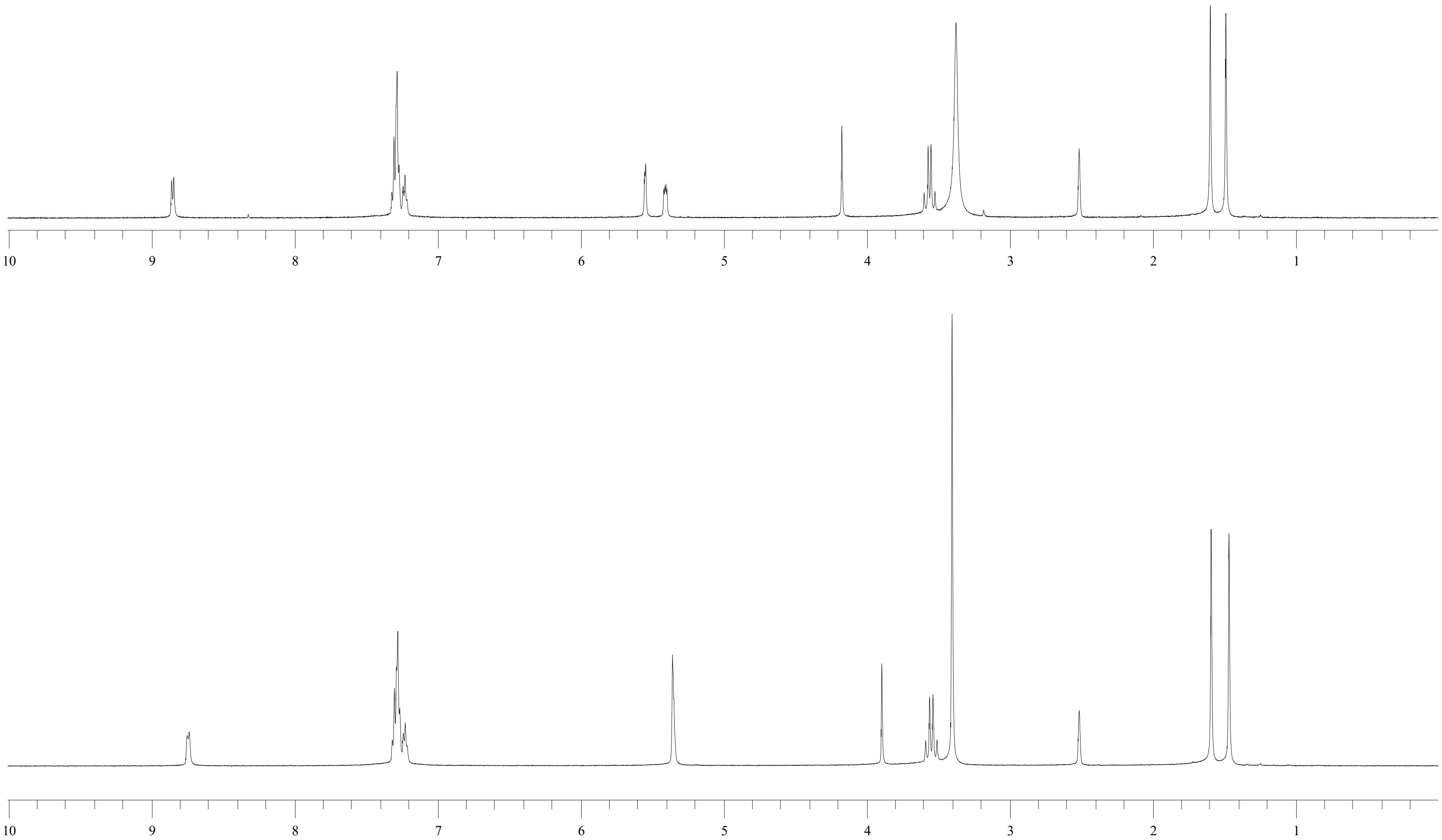


Figure S3. 1H-NMR spectra of **PenAg** (upper) and **PenNa** (down) in DMSO-$d_6$.

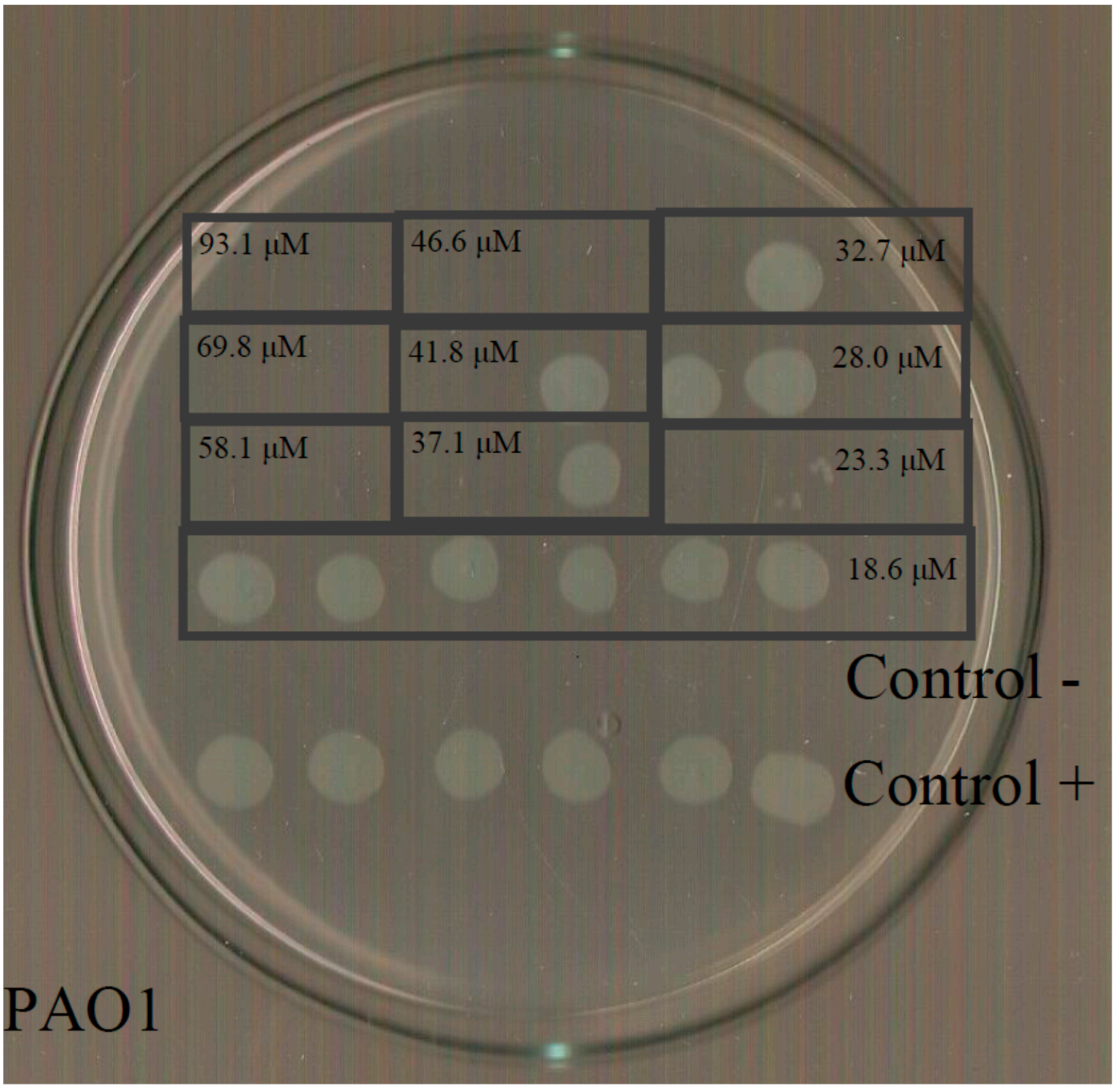


Figure S4. Minimum bactericidal concentration of **PenAg** against *P. auroginoza (PAO1)*.
